# Germinal matrix hemorrhage induces immune responses, brain injury, and motor impairment in neonatal rats

**DOI:** 10.1177/0271678X221147091

**Published:** 2022-12-22

**Authors:** Xiaoli Zhang, Jing Yuan, Shan Zhang, Wendong Li, Yiran Xu, Hongwei Li, Lingling Zhang, Xi Chen, Wenjun Ding, Jinjin Zhu, Juan Song, Xiaoyang Wang, Changlian Zhu

**Affiliations:** 1 Henan Key Laboratory of Child Brain Injury and Henan Pediatric Clinical Research Center, Third Affiliated Hospital and Institute of Neuroscience of Zhengzhou University, Zhengzhou, China; 2 NHC Key Laboratory of Birth Defects Prevention, Henan Key Laboratory of Population Defects Prevention, Zhengzhou, China; 3 Center for Perinatal Medicine and Health, Institute of Clinical Sciences, University of Gothenburg, Gothenburg, Sweden; 4 Center for Bran Repair and Rehabilitation, Institute of Neuroscience and Physiology, University of Gothenburg, Gothenburg, Sweden

**Keywords:** Germinal matrix hemorrhage, immune cells, chemokines, cytokines, motor dysfunction

## Abstract

Germinal matrix hemorrhage (GMH) is a major complication of prematurity that causes secondary brain injury and is associated with long-term neurological disabilities. This study used a postnatal day 5 rat model of GMH to explore immune response, brain injury, and neurobehavioral changes after hemorrhagic injury. The results showed that CD45^high^/CD11b^+^ immune cells increased in the brain after GMH and were accompanied by increased macrophage-related chemokine/cytokines and inflammatory mediators. Hematoma formed as early as 2 h after injection of collagenase VII and white matter injury appeared not only in the external capsule and hippocampus, but also in the thalamus. In addition, GMH caused abnormal motor function as revealed by gait analysis, and locomotor hyperactivity in the elevated plus maze, though no other obvious anxiety or recognition/memory function changes were noted when examined by the open field test and novel object recognition test. The animal model used here partially reproduces the GMH-induced brain injury and motor dysfunction seen in human neonates and therefore can be used as a valid tool in experimental studies for the development of effective therapeutic strategies for GMH-induced brain injury.

## Introduction

Germinal matrix hemorrhage (GMH) is a major complication of prematurity that can result in neonatal death, and survivors often suffer from neurological sequelae such as cerebral palsy (CP).^1^ GMH can cause subsequent brain injury such as periventricular leukomalacia or ventricular enlargement, and about half of infants with GMH-IVH (grades 3–4) develop CP.^2,3^ Currently, no widely accepted preventions or treatments are available for preterm GMH even though clinical study has shown promising result.^4,5^

The pathogenesis of GMH is multifactorial, and one important factor is the intrinsic fragility and vulnerability of blood vessels in the germinal matrix (GM).^6^ In addition, in the GM area, numerous vascularized glial and neuronal precursor cells accumulate and may disrupt the ependymal lining and extend into the lateral ventricle.^7,8^ The human GM reaches a maximum volume around 25 weeks of gestational age and progressively involutes into a residual mass present at 36 weeks of gestational age.^9^ Therefore, after this period of brain development, the incidence of GMH is uncommon. It has been reported that the GM in mice begins growing at E10.5, reaches its widest point at embryonic day 14.5, and involutes at the end of first week after birth.^10^ The rodent GM also contribute to the high susceptibility of the GM vasculature to hemorrhage.

Clearance of hematoma is a central pathophysiological process and contributes to immune response and inflammation in the brain and thus is one of the major causes of secondary brain injury after hemorrhage.^11–13^ Blood components and immune cells can also be independent factors for inducing/aggravating brain damage,^14^ and it has been shown that microglia/macrophage-mediated blood clot elimination occurs via PPAR‐γ stimulation and CD36 upregulation in GMH.^15^ However, the subtypes of immune cells and the role of preventing iron overload have not been clear in previous studies, and our understanding of the role of immune cells and inflammation in brain injury after GMH is still limited.

Magnetic resonance imaging (MRI) has well-documented advantages and reliability in detecting cerebral ischemic lesions, edema, brain hemorrhage, and white matter injury,^16,17^ and it has been used to measure volumetric and white matter injury by T2 sequencing and diffusion tensor imaging (DTI), respectively, in both clinical and preclinical studies.^18,19^ Using MRI, the rodent brain can be noninvasively and repeatedly interrogated, thus enabling detailed spatiotemporal mapping of brain injury within a single animal,^20^ and therefore MRI represents a good method for the evaluation of brain injury in animal models. However, there are a limited number of publications describing the use of MRI to assess brain hemorrhage and subsequent brain injury in preclinical models of GMH,^21,22^ and it is still unclear whether or not changes in brain pathology after hemorrhage in GMH models can be accurately evaluated and followed by MRI alone without additional histological assessments.

The purpose of the current study was to evaluate the dynamic changes in immune response in post hemorrhage brain injury over time and to evaluate neurobehavioral changes in the neonatal rat GMH model in order to develop potential neuroprotective and preventative strategies for this population of patients. We used postnatal day (PND) 5 rat pups because they are comparable to human fetus or newborn at 26–32 weeks gestation with respect to cortical developmental stages,^23^ the presence of the GM and the maturation of white matter.^24,25^ We found that CD45^high^CD11b^+^ immune cells and macrophage-related chemokines/cytokines and inflammatory mediators were increased in the brain after GMH, and this is corresponded to the appearance of hematoma and white matter injury as revealed by MRI. In addition, GMH rats showed mild abnormal motor function and locomotor hyperactivity that may indicate the clinical relevance of the current GMH model.

## Methods and materials

### Animals

Sprague–Dawley (SD) male and female rats aged 8–10 weeks were purchased from Vital River (Beijing, China) and were housed in a specific pathogen-free barrier animal facility at the Third Affiliated Hospital of Zhengzhou University. Animals were kept on a 12 h light/dark cycle and had free access to food and water. The pups were generated by crossing female and male rats and the animal experiments were carried out in compliance with FELASA guidelines and according to the 3Rs principle. The animal data reporting followed the ARRIVE 2.0 guideline.^26^ The inclusion criteria of the pups for this study were pups of both sexes with the body weight of 9–12 g at PND 5. The animals that died (mortality was 6.5%) during the experiments were excluded. Rats without hemorrhage in the brain after collagenases injection were excluded. A total 23 rat pups were excluded and a total of 133 pups (65 males and 68 females) were used for the analysis. The pups were randomly assigned to each group stratified by sex. The sample size was based our experimental design on numbers reported in previous studies.^25,27^

### GMH induction

**Figure 1.**
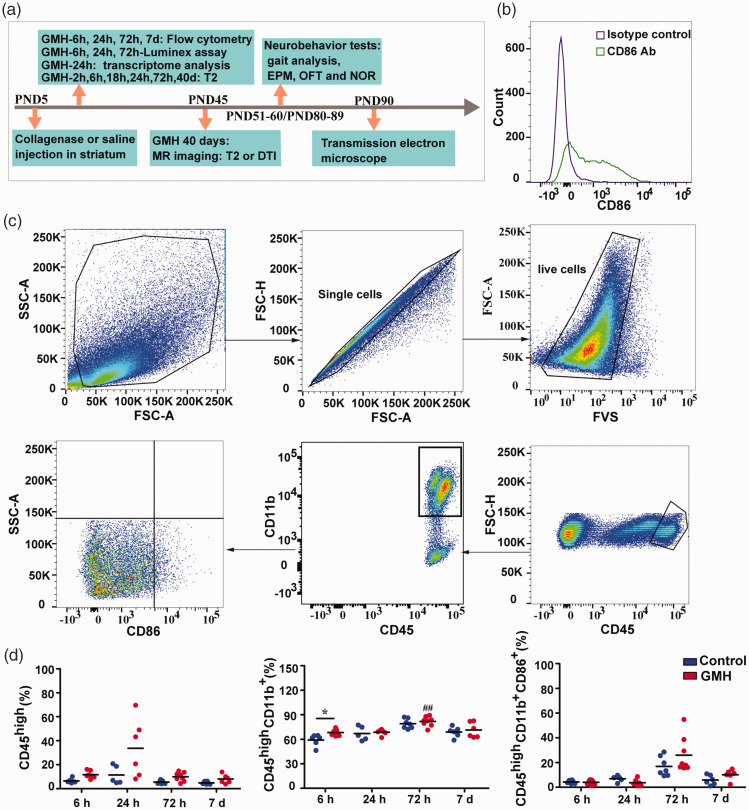
Immune cells infiltrated into the brain after GMH. (a) The study design. GMH was induced at postnatal day 5 by injection of collagenase. Some of pups were sacrificed at 6 h, 24 h, or 72 h for Luminex assay, some were sacrificed at 6 h, 24 h, 72 h, or 7 days for flow cytometry, and some underwent MRI and neurobehavioral tests prior to being sacrificed for transmission electron microscopy. (b) Isotype controls and antibodies for CD86 in the flow cytometry experiment. (c) The gating strategy for analyzing immune cells from the periphery and (d) The changes in immune cells (CD45^high^), immune cells (CD45^high^/CD11b^+^), and CD86 expression at 6 h, 24 h, 72 h, and 7 days after GMH in the brain. Data are shown as scatter plots of the mean values (6 h: n = 6 in control, n = 7 in GMH; 24 h: n = 5 in control, n = 6 in GMH; 72 h: n = 7 in control, n = 8 in GMH; 7 days: n = 6/group), and statistical analysis was performed using two-way ANOVA with Bonferroni post hoc test or Scheirer-Ray-Hare test. Control vs GMH: *p < 0.05, ***p < 0.001; GMH: 72 h vs 24 h or 6 h ^##^*p* < 0.01.

At PND 5, rat pups were randomly allocated into control and GMH groups. The grouping information was blinded to the persons running the experiments. Following anesthesia with isoflurane (5% for induction and 3.5% for maintenance) in a mixture of oxygen and nitrogen, 0.3 U collagenase VII (Sigma) in 2 µl saline or saline alone was injected into the right middle medial striatum for induction of GMH as previously described.^27^ Briefly, injections were administered at 1 μL/min for 2 min in the right hemisphere 1 mm rostral of the bregma and 4 mm lateral of the midline, and 3.5 mm in depth using a 28G needle and a 25 μL Hamilton syringe connected to an infusion pump (CMA/100 microinjection pump). The animals were allowed to recover on a heating pad set to 35°C before being returned to their dams. All animal experiments were approved by the Animal Care and Ethics Committees of The Third Affiliated Hospital of Zhengzhou University (ethical number: 2021-028-01). The study design is shown in Figure 1(a).

### Protein extraction and Luminex assay

The rats were sacrifices, and their brains were collected at 6 h, 24 h, and 72 h after injection and then stored at −80°C. The entire ipsilateral hemisphere to the collagenase injection was used for analysis. The brains were thawed, added to 250 µl cell lysis buffer containing 10 mM EDTA, 2% Triton X-100, and 2% protease inhibitor cocktail (Sigma P8340), and homogenized in a small tissue breaker. The protein content was determined using the bicinchoninic acid assay (C503051, Sangon Biotech, China), with bovine serum albumin (BSA) as the standard according to the manufacturer’s protocol. Sample absorbance was read at 560 nm using a Spark 10 M (Tecan, Switzerland). All samples were assayed in duplicate for rat chemokines and cytokines using the Rat Chemokine/Cytokine Panel multiplex kit (Cat#EPX220-30122-901, eBioscience) and Luminex 200 (Nasdaq, USA).

### Flow cytometry analysis

Rats were transcardially perfused with ice-cold phosphate-buffered solution (PBS) at 6 h, 24 h, 72 h or 7 days after GMH induction. The injured brains were quickly removed and cut into pieces and the Neural Tissue Dissociation Kit (Cat#130092628, Miltenyi Biotec) was used to acquire a single cell suspension using a MACS Octo Dissociator with heaters. After centrifugation at 500 × *g* for 5 min at room temperature, the cells were re-suspended in 30% Percoll and 70% Percoll (GE Healthcare). After centrifugation at 1000 × *g* for 30 min at room temperature, mononuclear cells were at the mid-layer between the 30% and 70% Percoll interface. Cells were purified by washing with FACS buffer (0.5% BSA in PBS) twice through a 70 µM cell strainer (BD Biosciences). To determine the extent of cell death, FVS-BV510 (BD Biosciences) was added and incubated for 15 min. Mononuclear cells were stained with anti-rat CD11b-FITC (WT.5, BD Biosciences), anti-rat CD45-PE-Cy5 (OX-1, BD Biosciences), and anti-rat CD86-BV421 (24F, BD Biosciences) following standard protocols and the manufacturer’s instructions. Data were obtained using a FACS Canto II (BD Biosciences) and analyzed with Flow Jo 10.0 software.

### Transcriptome analysis of brain tissue

Our previous GMH datasets (BioProject ID: PRJNA756842) were collected from the BioSample database and the timepoint was 24 h after GMH induction,^27^ and the differentially expressed genes (DEGs) were identified using the limma, deseq2, and edegR packages in R (4.1.0). The resulting *p*-values were adjusted using the Benjamini and Hochberg approach for controlling the false discovery rate. Genes with an adjusted p-value <0.05 were assigned as DEGs, and the RNAseqStat2 software was used for KEGG pathway enrichment analysis (RNAseqStat2: A Pipeline to Process RNAseq Data (https://github.com/xiayh17/RNAseqStat)).

### MRI

MRI scans were performed at 2 h, 6 h, 18 h, 24 h, 72 h, and 40 days after GMH induction with a preclinical MR scanner 4.7T (MR solution, United Kingdom) with a gradient strength (x, y, and z) of 600 mT/m. Multislice, T2w, and images were acquired and reconstructed using Preclinical Scan v1.2 (MR Solution). The DTI scanning were performed at 40 days after GMH. Images were acquired and reconstructed using Preclinical Scan v1.2, EPI recon, DSI studio, and FSL. The brain atlas was obtained from the literature.^28^

### CatWalk automated quantitative gait analysis

For all behavior tests, there were 17 rats (male = 6, female = 11) in the GMH group and 17 rats (male = 9, female = 8) in the control group. We conducted two batches of behavior tests, first in PND 50-60, and the second in PND 80–89 (Figure 1(a)). The rats were placed in the room at 15:00 in the afternoon for a period of 30 min for acclimatization on the day prior to the tests. The *CatWalk* XT automated gait analysis system (Noldus Information Technology, Wageningen, The Netherlands) consists of a horizontal glass plate 1.3 m in length covered by a removable tunnel creating a dimmed light on the walkway. The animals are positioned at the beginning of the walkway and voluntarily traverse the plate towards their home cage. A green LED light is emitted inside the glass plate and is internally reflected, except for those areas where contact with the glass plate is made. If the paws touch the glass, light is refracted on the opposite side. These illuminated areas are automatically detected by a high speed color camera that is positioned underneath the glass plate. The data are sent to a computer and automatically analyzed by the *CatWalk* XT software. After successive trainings for five days (from PND 46 to 50), the animals had to perform a minimum of three nonstop runs to qualify for *CatWalk* XT analysis on the test day at PND 51. A maximum speed variation of 60%, a camera gain of 23.4 dB, and a detection threshold of 0.12 were set for the detection of all parameters used in the experiments.

### Elevated plus maze (EPM)

At PND 58, the EPM test was performed using an acrylic glass maze comprising a central part (10 cm × 10 cm), two opposing open arms (50 cm), and two opposing closed arms (50 cm). The apparatus was set to a height of 50 cm, and the open arms were provided with uniform overhead illumination of 5 lux. Rats were placed in the open arm point close to center facing the closed arms, and video recordings were made for a total duration of 5 min.

### Open field test (OFT)

At PND 59, the OFT was performed in a black acrylic glass box (100 cm × 100 cm × 40 cm) with an overhead lamp directed to the center of the field. Each rat was placed in the corner of the apparatus, and locomotion parameters were recorded for 10 min.

### Novel object recognition (NOR)

At PND 60, the NOR test was used to assess recognition memory in the rats after the GMH operation. The NOR test consisted of three parts: habituation, training/object familiarization, and NOR testing, and there was a lapse of one hour between training and testing. NOR was carried out in the same box used for the OFT. The recognition index is the proportion of time spent with the novel object compared to the total exploration time for both objects.

### Transmission electron microscopy (TEM)

Rats that have showed white matter damage by MRI were used for TEM (n = 3 for GMH and n = 2 for control). These rats were transcardially perfused with saline after behavioral tests and the brain samples were fixed in 2.5% glutaraldehyde solution in 0.1 M PBS. Tissue blocks of 1 mm^3^ were taken from the external capsule and rinsed with 0.1 M phosphoric acid buffer (pH 7.0) three times. The samples were fixed with 1% osmium solution and dehydrated with an ethanol gradient and then treated with a series of pure acetone, a 1:1 mixture of embedding agent and acetone, a 3:1 mixture of embedding agent and acetone, and finally embedding agent alone. The embedded samples were heated at 70°C overnight, sliced on a Leica EM UC7, stained with lead citrate solution and uranium dioxyacetate, and observed under a transmission electron microscope (Gemini SEM 360). Images per animal were acquired at 2000×magnification.

### Data analysis

Data were analyzed with SPSS 21.0 (IBM, USA). All data were tested for normality by the Shapiro–Wilk test before the subsequent statistical analysis. Homogeneity of variance of the data was tested by Levene’s test. To compare the control and GMH groups, we used a two-tailed unpaired t-test or Mann–Whitney U-test. For the data in the control and GMH groups at different timepoints, two-way ANOVA with the Bonferroni post hoc test or the Scheirer–Ray–Hare test, a nonparametric test used for two-way factorial designs, were used for the analysis.^29^ Gene expressions data (FPKM) produced by RNAseq were analyzed by the DEseq2 software package that is designed for normalization, visualization, and differential analysis of high-dimensional count data. *p*-values <0.05 were considered statistically significant.

## Results

### GMH caused early immune cell infiltration into the brain

The involvement of immune cells in GMH-induced damage was detected at early time points by flow cytometry (Figure 1(b) and (c)). Overall, similar immune cell populations were found in the brains of both control and GMH rats. However, the proportion of specific inflammatory cell types differed in GMH rats compared with controls. CD45 is a transmembrane protein tyrosine phosphatase located on most hematopoietic cells,^30^ and the appearance of CD45^high^ cells in the brain suggests that they are likely immune cells that infiltrate from the periphery, or are activated brain-resident immune cells such as activated microglia.^31–33^ CD11b, the α-chain of the integrin CD11b/CD18 is expressed on the surface of infiltrating leukocyte such as macrophages and neutrophils, as well as microglia in the brain, and it modulates several biological functions including cell adhesion, migration, and signaling.^34,35^ There were no significant CD45^high^ cell increase at any timepoints (6 h, 24 h, 72 h or 7 days) post GMH in the GMH brains compared to controls (H_1,51_ = 3.65, *p* = 0.06, main effect of injury; *p* < 0.05 for the statistical test for homogeneity of variance, Figure 1(d)). There was no difference in CD45^high^ cells between the different time points (H_3,51_ = 7.18, *p* = 0.07, main effect of time), and no interaction effect was found for time and injury in CD45^high^ cells (H_3,51_ = 2.63, *p* = 0.45, interaction effect of time and injury).

In addition, we observed an increase in CD45^high^/CD11b^+^ immune cells (*p* = 0.015) at 6 h in GMH brains compared to controls (F_1,51_ = 4.73, *p* = 0.03, main effect of injury; *p* = 0.054 of statistical test for homogeneity of variance, Figure 1(d)). When comparing different time points, CD45^high^/CD11b^+^ immune cells also significantly increased and peaked at 72 h in both saline and GMH groups (F_3,51_ = 17.44, *p* < 0.001, main effect of time, Figure 1(d)). In detail, the proportion of CD45^high^/CD11b^+^ immune cells at 72 h was higher than at 6 h (*p* < 0.01) and 24 h (*p* < 0.01) in GMH groups. However, no interaction effect was found for time and injury in CD45^high^/CD11b^+^ cells (F_3,51_ = 0.91, *p* = 0.44, interaction effect of time and injury).

CD86 is expressed on the antigen presenting leukocytes and activated microglia and acts as a pro-inflammatory factor.^36,37^ In the brain, the proportion of CD45^high^/CD11b^+^/CD86^+^ cells did not change at any timepoint post-GMH compared to the controls and did not varied as time passed in both control and GMH groups (H_3,51_ = 7.24, *p* = 0.06, main effect of time; H_1,51_ = 0.01, *p* = 0.92, main effect of injury; *p* < 0.05 in the statistical test for homogeneity of variance, Figure 1(d)). No interaction effect was found between time and injury on CD86 expression in CD45^high^/CD11b^+^ cells (H_3,51_ = 1.02, *p* = 0.80, interaction effect of time and injury).

### GMH caused early chemokine and cytokine responses

**Figure 2.**
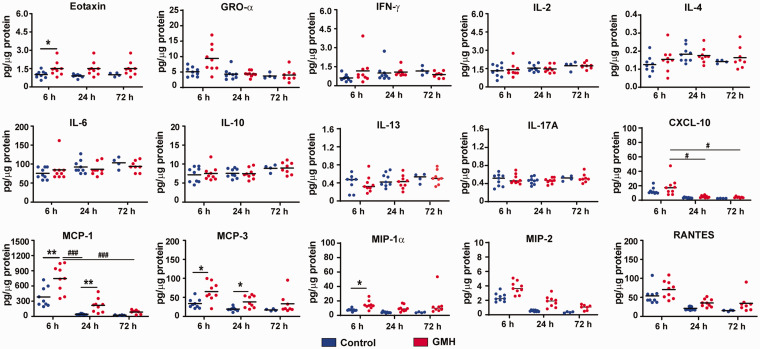
Cytokine and chemokine levels after GMH. The bar graphs show the quantification of cytokines and chemokines in brain tissue at 6 h, 24 h, and 72 h after GMH. Data are shown as scatter plots of the mean values (6 h and 24 h: n = 9/group; 72 h: n = 4 in control group, n = 8 in GMH group). Statistical analysis was performed using two-way ANOVA with Bonferroni post hoc test or Scheirer-Ray-Hare test with Kruskal-Wallis for pairwise comparison *p < 0.05, **p < 0.01, ***p < 0.001. **^#^** *p* < 0.05, **^##^** *p* < 0.01, **^###^** *p* < 0.001.

To further understand the immune response elicited by GMH, brain samples were also analyzed for chemokines and inflammatory cytokines (Figure 2), and in total 22 chemokines and cytokines were measured. Among them, eotaxin was significantly upregulated in the GMH group compared to controls (F_1,48_ = 4.39, *p* = 0.04, main effect of injury, *p* = 0.145 for the statistical test for homogeneity of variance) and the level of eotaxin was higher (*p* = 0.03) at the 6 h timepoint in the GMH group compared to the control group. Eotaxin did not change as time passed in either the control or the GMH group (F_2, 48_ = 2.10, *p* = 0.14, main effect of time). No interaction effect was found in the GMH group or control group in terms of eotaxin levels (F_2, 48_ = 0.89, *p* = 0.42).

The macrophage-related chemokine MCP1 increased after GMH induction (H_1, 48_ = 4.20, *p* = 0.04, main effect of injury), and it decreased as time passed (H_2, 48_ = 9.64, *p* = 0.01, main effect of time). No interaction effect of injury and time was found for MCP1 (H_2, 48_, *p* = 0.28). MCP1 was significantly higher (*p* = 0.004) in GMH rats at 6 h but not 24 h and 72 h post GMH (*p* < 0.05) compared to controls, and in the GMH group. MCP1 decreased at 24 h (*p* < 0.001) and 72 h (*p* < 0.001) compared with 6 h. MCP3 was elevated in the brain tissue after GMH (H_1, 48_ = 2.98, *p* = 0.04, main effect of injury) and it was significantly higher at 6 h (*p* = 0.01) and 24 h (*p* = 0.03) in GMH rats compared to controls. No effect of time was found in either the GMH or control groups (H_2, 48_ = 2.98, *p* = 0.22, main effect of time) and no interaction of time and injury on the level of MCP3 (H_2, 48_ = 0.29, *p* = 0.86). MIP-1α expression was increased after GMH (H_1, 48_ = 4.22, *p* = 0.04, main effect of injury), but the difference was only seen at 6 h (*p* = 0.045) and not at 24 h or 72 h (*p* < 0.05). The level of MIP-1α did not change with time (H_2, 48_ = 1.01, *p* = 0.60, main effect of time), and there was no the interaction of time and injury (H_2, 48_ = 0.30, *p* = 0.86). MIP2 levels did not change significantly after GMH and time (H_1,48_ = 3.30, *p* = 0.06, main effect of injury; H_2, 48_ = 4.17, *p* = 0.12).

No significant changes in RANTES were seen between the GMH and control groups (H_1,48_ = 1.33, *p* = 0.99, main effect of injury) although the level changed with time (H_2, 48_ = 6.10, *p* = 0.047, main effect of time). The RANTES level decreased at 24 h (*p* < 0.05) and 72 h (*p* < 0.05) compared to 6 h in both control and GMH groups. Injury did not cause changes in CXCL-10 between the GMH and control groups (H_1,48_ = 1.14, *p* = 0.28, main effect of injury). The level of CXCL-10 at 6 h (*p* < 0.05) was higher than at 24 h and 72 h in the GMH group (H_2, 48_ = 9.11, *p* = 0.01, main effect of time). No interaction effect was found for CXCL-10 (H_2, 48_ = 0.30, *p* = 0.86).

No significant differences were found for the levels of GRO-α, INF-γ, IL-2, IL-4, IL-6, IL-10, IL-13, or IL-17A at 6 h, 24 h, or 72 h between the control and GMH groups. Other cytokines, such as IL-5, TNF-α, G-CSF, GM-CSF, IL-1α, IL-1β, and IL-12p70, were below the detection level or were at very low levels (data not shown).

### The GMH-induced inflammatory response was dominated by monocytes

**Figure 3.**
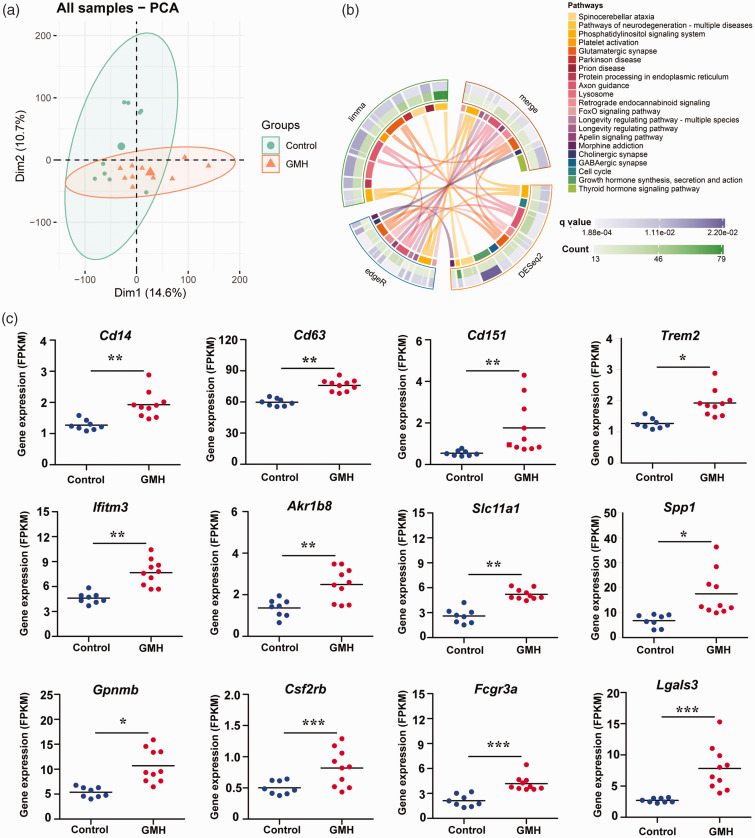
Monocyte/macrophage-related gene expression and involved pathways at 24 h after GMH. (a) Principal component analysis showing the variation between control and GMH samples. (b) The involved pathways were counted in the circles and linked by lines. DEGs identified by three different statistical methods (limma, edgeR, and DESeq2) were separately analyzed for KEGG pathway enrichment and (c) The expression of selected monocyte/macrophage-related genes in the control and GMH groups. Data are shown as scatter plots of the mean values (n = 8 in control group, n = 10 in GMH group). *p < 0.05, **p < 0.01, ***p < 0.001.

We further evaluated the expression pattern of selected genes related to monocytes/macrophages^38^ in control and GMH brains at 24 h after injury. According to principal component analysis of the data, both groups could be differentiated from each other (Figure 3(a)). KEGG analysis showed that these monocyte/macrophage-relevant genes were enriched in the pathways of neurodegeneration, axon guidance, and synapse processes (Figure 3(b)). When comparing the individual gene expression levels in GMH *vs.* controls, we found that multiple genes were elevated in the GMH rat brains, including the low-expression gene *Csf2rb*, the medium expression genes *Cd14*, *Cd151*, *Trem2*, *Ifitm3*, *Akr1b8*, *Slc11a1*, *Fcgr3a*, and *Lgals3*, and the high-expression genes *Cd63*, *Spp1*, and *Gpnmb* (Figure 3(c)).

### Characteristics and temporal changes of hemorrhage and brain injury after GMH

**Figure 4.**
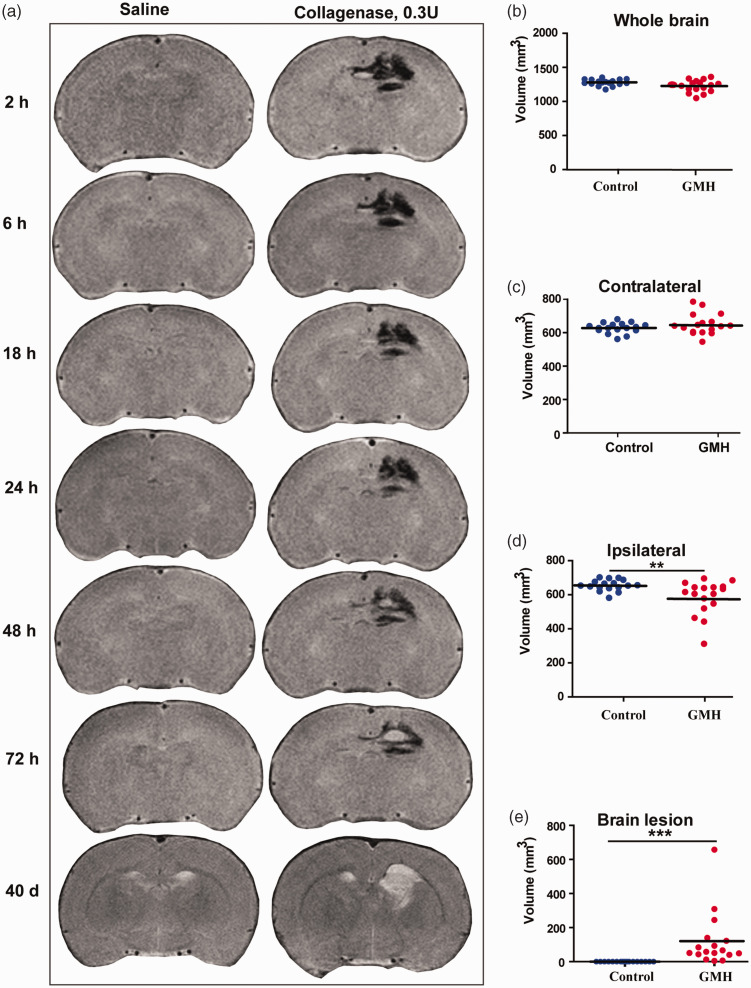
Temporal changes in the T2 signal in the brain and brain volume at 40 days after collagenase injection. (a) Representative cerebral coronal MRI images at 2 h, 6 h, 18 h, 24 h, 48 h, 72 h, and 40 days after saline or collagenase injection. (b) The total brain volume. (c) The volume of the contralateral hemisphere (d) The volume of the ipsilateral hemisphere and (e) The injury volume. Data are shown as scatter plots of the mean values (n = 17/group, male = 6, female = 11 in GMH group; male = 9, female = 8 in control group), two-tailed unpaired t-test or Mann-Whitney U test, **p < 0.01.

Following collagenase injection, hematoma in the brain was obvious as early as 2 h post injection and remained stable for 24 h after collagenase injection. Subsequently, high T2 signal, indicating deoxygenated hemoglobin, began to appear at 48 h in the center of the hematoma and gradually enlarged at 72 h. Up to day 40, the injured brain area still showed high T2 signal (Figure 4(a)). No difference in total brain volume was found in the GMH group compared to controls at day 40 after hemorrhage induction (homogeneity test of variances: F = 5.17, *p = *0.03; t = 2.04, *p* = 0.52. Figure 4(b)). However, the volume of the right (ipsilateral) hemisphere after GMH was significantly smaller than controls (*p < *0.001) but not the left (contralateral: homogeneity test of variances: F = 4.60, *p = *0.04; t = −1.72, *p* = 0.09) hemisphere (Figure 4(c) and (d)), and the total brain lesion volume was significantly higher than in the control group (*p* < 0.001) (Figure 4(e)).

### GMH induced white matter structural changes and axon loss

**Figure 5.**
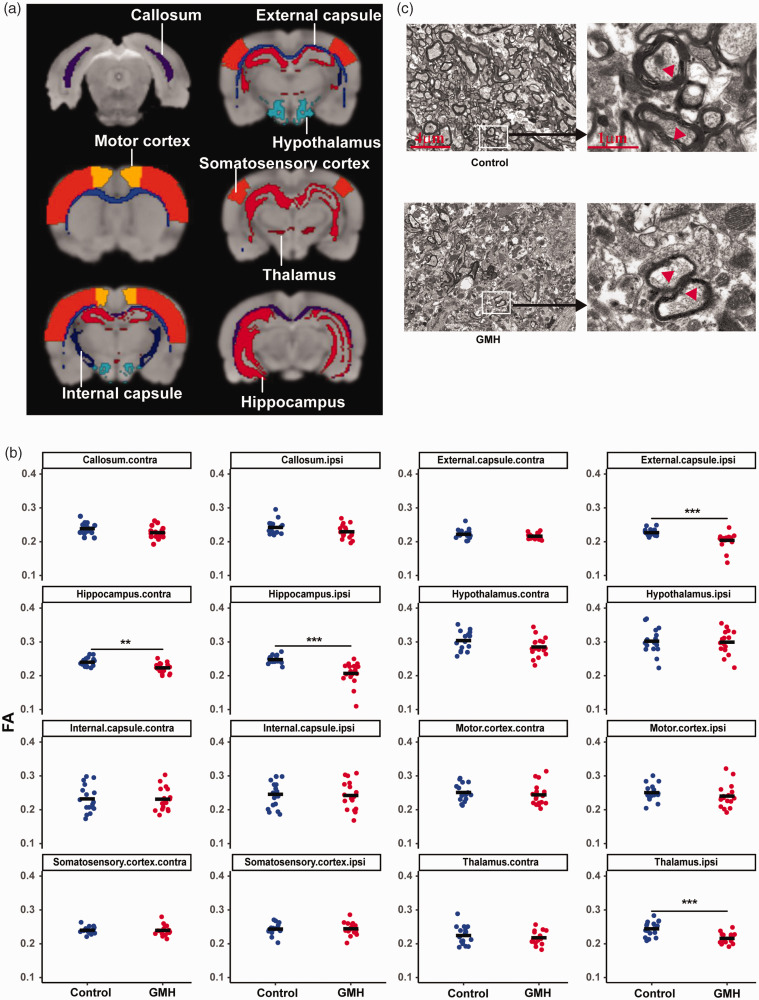
GMH-induced microstructure changes in the white matter. (a) The representative brain regions of interest: corpus callosum, external capsule, internal capsule, hippocampus, hypothalamus, motor cortex, somatosensory cortex, and thalamus. (b) FA by DTI scanning in the regions of interest at 40 days after GMH between the control and GMH groups. Data are shown as scatter plots of the mean value (n = 17/group) and (c) Representative electron microscopy images of the external capsule of control. Data are shown as scatter plots of the mean values, two-tailed unpaired t-test. **p < 0.01, ***p < 0.001.

To explore the white matter structural changes after GMH, the regions of interest for the structural connectivity analyses were projected onto a T2 map in MRI. The fractional anisotropy (FA) values were measured by DTI on day 40 after collagenases injection in the following white matter structures: corpus callosum, external capsule, internal capsule, hippocampus, hypothalamus, motor cortex, somatosensory cortex, and thalamus (Figure 5(a)). The GMH group showed decreased FA in the ipsilateral external capsule (*p* = 0.001), contralateral hippocampus (F = 0.24, *p* = 0.63; t = 3.49, *p = *0.001), ipsilateral hippocampus (F = 6.19, *p* = 0.02; t = 4.71, *p* < 0.001), and ipsilateral thalamus (F = 2.10, *p* = 0.16; t = 4.36, *p* < 0.001) compared with controls (Figure 5(b)). There were no significant differences in the other brain areas between the GMH rats and controls.

We further examined the ultrastructural changes in the external capsule white matter by TEM at PND90 (Figure 5(c)) to confirm the white matter injury. For this, we only selected the rats that had identified white matter damage in the external capsule based on MRI or DTI results. The axons and myelin sheaths in the control group showed normal structures, while the GMH rats displayed obvious pathological changes, including axon loss and damaged myelin sheaths, which were characterized by structural de-arrangement and thinner myelination (Figure 5(c)).

### GMH induced motor dysfunction

**Figure 6.**
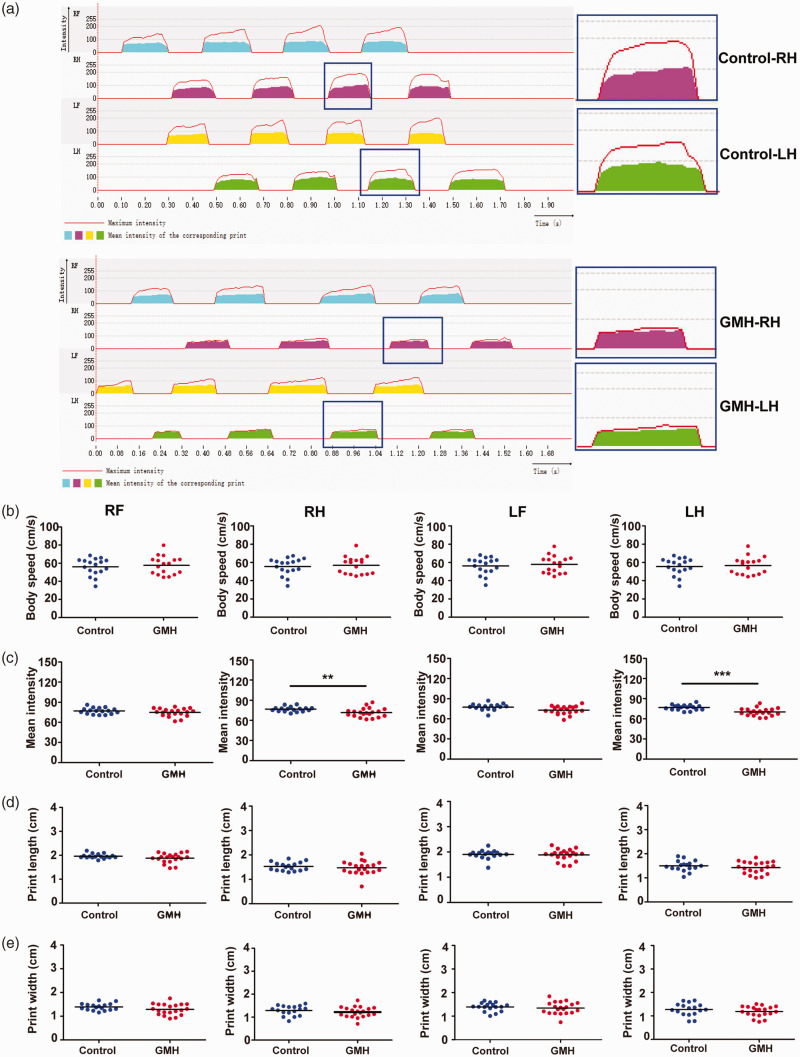
GMH-induced motor dysfunction. (a) Representative images of the 2D footprint intensities of the four paws in the gait analysis and enlarged images for the hind paws of control and GMH rats. (b) Body speed. (c) Mean intensity. (d) Print length and (e) Print width. Data are shown as scatter plots of the mean values (n = 17/group), two-tailed unpaired t-test, *p < 0.05, **p < 0.01, ***p < 0.001. RF: right front limb; RH: right hind limb; LF: left front limb; LH: left hind limb.

Next, we asked whether GMH causes behavioral changes in rats. First, rats were examined using the *CatWalk* XT system to analyze the walking pattern by recording the footprints (Figure 6(a)). The body speeds were similar in the GMH and control groups (Figure 6(b)). However, compared to control animals the mean intensity of the footprints was significantly reduced in the right hind paws (F = 4.08, p = 0.05; t = 2.78, p = 0.009) and left hind paws (F = 1.51, *p* = 0.23; t = 4.09, *p* < 0.001) after collagenase injection (Figure 6(c)). Print width and print length are static parameters of the paws in gait analysis, and no significant changes in these two parameters were found, although there was a downward trend after collagenase injection (*p* > 0.05, Figure 6(d) and (e)). Other parameters showed no significant difference between the control and GMH groups (data not shown).

### GMH did not affect anxiety-like behaviors or recognition function

**Figure 7.**
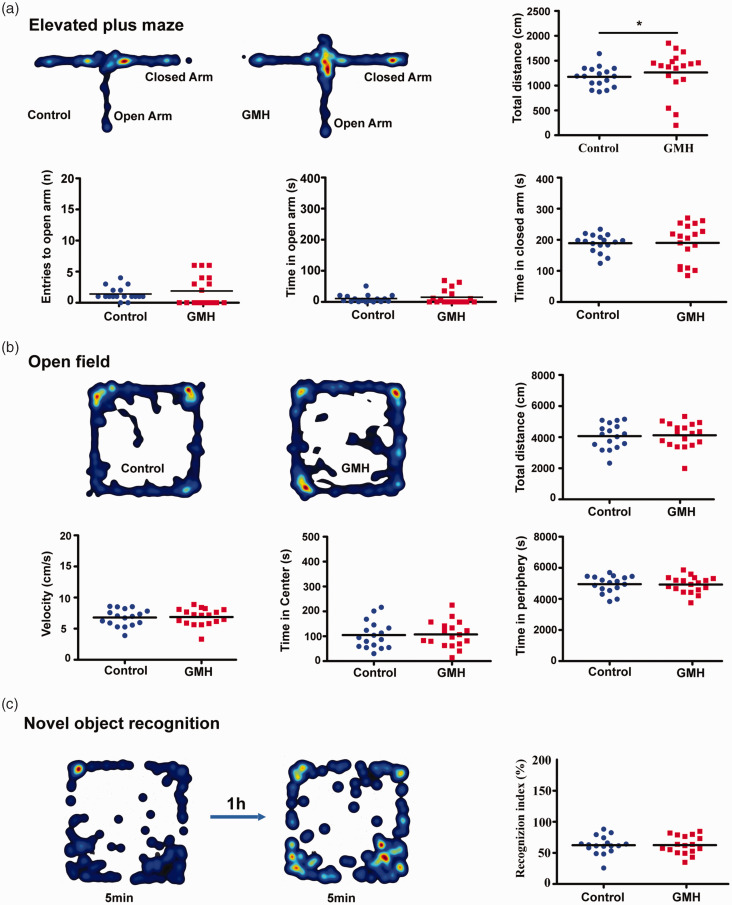
Neurobehavioral assays after GMH. (a) Heat maps illustrating the proportion of time spent in each type of arm in the control and GMH rats. The EPM was used to measure anxiety. The total distance covered, entries into the open arm, time spent in the open arms, and time spent in the closed arms. (b) The OFT was used to quantify locomotion, velocity, thigmotaxis (time spent in the periphery), and time spent in the center of the arena. The heat maps represent the proportion of time in the center or periphery zones of arena and (c) The NOR test was used to measure memory function. The heat maps show the time spent with familiar or novel objects. Data are shown as scatter plots of the mean values (n = 17/group, male = 6, female = 11 in GMH group; male = 9, female = 8 in control group), two-tailed unpaired t-test or Mann-Whitney U test, *p < 0.05.

To determine the effect of GMH on anxiety, we measured anxiety-like behavior and locomotor activity in the EPM test. GMH animals showed increased total distance compared to control animals (*p* = 0.047). However, there were no differences in entries to the open arm (*p* = 0.089), time spent in the open arm (*p* = 0.37) or closed arm (*p* = 0.55) between GMH and control rats (Figure 7(a)).

The OFT was used to test locomotor activity by total traveled distance (cm/10 min) and velocity (cm/s) in rats after collagenase injection. No differences were found between the control and GMH groups regarding the total distance (F = 0.036, *p* = 0.85; t = −0.49, *p = *0.62), velocity (F = 0.049, *p* = 0.83; t = −0.49, *p* = 0.63), or time spent in the center (F = 0.001, *p* = 0.98; t = −0.37, *p* = 0.71) or in the periphery (F = 0.004, *p* = 0.95; t = 0.37, *p* = 0.72) (Figure 7(b)). NOR was used to evaluate recognition memory based on the tendency of rats to discriminate between familiar objects and new objects. No significant changes in the recognition index (F = 1.20, *p* = 0.28; t = −0.51, *p* = 0.62) were found between the control and GMH groups (Figure 7(c)). To note the second batch of neurobehavior tests did not show any positive results, and all observed neurobehavioral deficits were recovered observed by PND80-90 (data not shown).

## Discussion

In this study, we show the spatiotemporal changes of hemorrhagic brain injury induced by collagenase injection in rats. We found that CD45^high^CD11b^+^ immune cells increased in the brain at early time points after GMH, and this was accompanied by increased macrophage-related chemokines/cytokines and inflammatory mediators in the brain tissue. In addition, rats with GMH injury showed mild abnormal motor function and locomotor hyperactivity, indicating the clinical relevance of this model.

GMH has been modeled in several species such as rodents, rabbits, sheep, cats, and dogs, and it has been found that hemorrhage triggers robust inflammation around the cerebral ventricles, damages axons, and induces both apoptosis and maturational arrest of oligodendrocyte progenitors, thus leading to reduced myelination of the white matter.^1,21^ The lesions caused by GMH were characterized by a combination of neuronal, axonal, and glial loss or hyperplasia, at the peri-lesion sites. Microtubule associated protein-2, myelin basic protein, Iba-1 and GFAP were often used as markers for evaluating the brain lesions. It was reported in PND7 rat collagenase GMH models that neuron cell loss, microglia activation and reactive astrogliosis and myelin loss were cytopathological mechanisms after GMH.^15,39,40^ Similarly, smaller brain volume, white matter injury and astrogliosis were observed after GMH induction in a PND5 GMH rat model.^25^ In addition, ferroptosis and mitochondrial dysfunction were found at early time points after GMH in the PND5 GMH rat model.^27^

Brain injury after GMH includes the acute physical effects of bleeding^14^ and the secondary injury arising from heme and free iron-induced cytotoxicity and regulated neural cell death, which can persist for a long time and are associated with long-term neurological disabilities and thus have attracted much attention in the study of post-hemorrhagic brain injury mechanisms. It has been shown that even low-grade GMH may impair normal oligodendrogenesis thus leading to white matter injury,^41^ and immune cell infiltration and inflammation have been shown to play important roles in GMH-induced secondary brain injury.^14^ In this study, we confirmed that macrophages may infiltrate into the injury area within 6 hours after induction of GMH in neonatal rats. We observed increases in macrophage chemoattractant proteins (MCP-1 and MCP-3) and macrophage inflammatory proteins (MIP-1α and MIP-2), which was similar to a rabbit model of GMH that found an increase in the mRNA levels of MCP1 and IL-8 in the choroid plexus.^42^ Other studies showed that peripheral immune cells (MPO^+^ cells) and microglia infiltrated into the injured area in rabbit and rat pups after GMH,^25,43,44^ suggesting that inhibition or knockout of MCP-1 or its receptor might be protective against the development of brain injury.^45^ These results suggest that blockage of monocyte-related chemokines might be an important strategy for the treatment of neonatal GMH. To note, in the current study we did not find any significant differences for GRO-α, INF-γ, IL-2, IL-4, IL-6, IL-10, IL-13, or IL-17A at any time point between GMH and control animals. This might be at least partly due to the fact that the entire hemispheres that is ipsilateral to the collagenase injection was used for Luminex analysis.

In addition to monocyte/macrophage-related chemokines, we also found that the CD45^high^/CD11b^+^ population increased at early time points after injury. The CD45^high^/CD11b^+^ cell population plays an important role in phagocytosis and in the monocyte/macrophage composition of various subpopulations with or without CCR2 expression,^46,47^ and we observed at least two clusters of CD45^high^/CD11b^+^ populations involved in post-hemorrhagic brain injury. It has been shown that CD45^high^/CD11b^+^ cells appear in the injured area and express high levels of Trem2, CD11c, and several disease-associated microglia signature genes and have high phagocytic capacity,^48^ which suggests that this cell population might be involved in hematoma scavenging after hemorrhage.

The CD45^high^CD11b^+^ immune cell population might consist activated microglia because microglia have been shown to change phenotype from CD45^mediate^ CD11b^+^ to CD45^high^CD11b^+^ and express *Spp1 and Lgals3* in brain development and in disease pathology^32,33,49^ and they showed phagocytic ability thus sharing a disease associated microglia gene signature and indicating potential role in the pathological process of GMH. In addition, CD45^high^/CD11b^+^/Ly6G^−^ cells can differentiate into osteopontin-producing galectin-3^high^/CD206^+^ macrophages, and galectin-3 is essential for transcriptional activation of osteopontin, and these macrophages thus play an important role in tissue repair after acute injury.^50^ In the current study, *Spp1*and *Lgals3* levels were clearly changed, and elevated osteopontin levels were also observed in the injured white matter of postmortem brains from preterm infants with GMH.^51^ In this model, the identification and characterization of these CD45^high^/CD11b^+^ cells and their relationship with *Spp1* and *Lgals3*, which may play either a neuroprotective or damaging role after brain injury,^50,52^ need further study.

MRI can provide a more detailed visualization of the location and extent of brain hemorrhage and thus can help predict adverse outcomes in preterm infants.^53^ After intracerebral hemorrhage, hemoglobin changes from oxyhemoglobin to deoxyhemoglobin and hemosiderin and iron are released, which can be detected by MRI.^54^ It has been found that cognitive impairments in stressed rats correlate with various DTI metrics of the corpus callosum and amygdala,^55^ and experience-induced differences in white matter and grey matter structures have also been observed by DTI in adult rats.^56^ In this study, we first used DTI to evaluate the different brain regions after hemorrhage. Not only the subcortical white matter, but also the white matter in the thalamus distant from the hematoma was damaged, which advances our understanding of white matter damage in this model. Beyond this, new MRI sequences and improved hardware will continue to help us to understand hemorrhage and hematoma, and their anatomical locations at acute stages could be the main prognostic markers for cerebral hemorrhage outcome.^57^

GMH-induced white matter injury is the most important risk factor for long-term neurologic sequelae such as CP, cognitive deficits, and learning impairments.^1^ Experimental studies with GMH models have shown motor impairments using rotarod or foot fault tests, learning and memory impairments using a water maze, and hyperactivity using the OFT.^25,58,59^ In addition, gait abnormalities can be found in preterm brain injury rodent models such as hypoxia ischemia or LPS-induced brain injury models.^60,61^ Impaired gait constitutes a crucial functional limitation in patients with CP, and we found that the mean intensity of paw prints was lower in the neonatal rat GMH model, which indicated that rats became lame after GMH and that this model might be possible to use for evaluation of preventive strategies against CP. It was worth noting that the gait abnormalities and other neurobehavior abnormalities in the model were mild. This may partly be due to the fact that the capacity for restoration of function after brain injury in rodent animals is strong.^61–63^ This is further confirmed in the current study that no positive results were obtained in our second batch of neurobehavior tests conducted later between PND 80 and PND 89.

The OFT is often used for locomotor activity and anxiety-like emotion in animal models of diseases,^64^ and one study showed that a difference was found between control and GMH rats after 10 minutes in the arena of the OFT,^25^ which indicated that the time in our experiment might not have been long enough, although trial lengths can be as short as 2 min and up to an hour in the OFT.^65^ The EPM relies on the rodent’s exploratory nature and is a better test than the OFT for detecting anxiety in rodents,^64,65^ and no differences were found between GMH and control rats in the present study in the EPM test, except that an increased total distance traveled in GMH rats was found. In EPM, the anxiety is expressed by the animal spending more time in the enclosed arms which we did not observe in the current study. However, the total distance traveled was considered a quantitative evaluation of locomotor activity. Thus our results are consistent with previous observations using the same GMH model.^25^ Locomotor hyperactivity is a core to the construct of attention deficit hyperactivity disorder. Preterm brain injury including GMH, is associated with an increased risk of neurodevelopmental disorders.^66^ Therefore the fact that GMH rats showed locomotor hyperactivity again confirmed the clinical relevance of the current GMH model.

In addition, we did not find any learning or memory impairments in our study. There are two possible reasons for the lack of a difference in the above neurobehavioral tests in the neonatal rat GMH brain injury model. First, the age of the rats in our GMH model and the time of the behavioral evaluation were different from other studies. Some of them used PND 7 rodents to induce the GMH model or evaluated anxiety or motor-behavior of rodents at day 22 to 40 day after GMH.^25,59^ Rodents have a strong capability for neurobehavior recovery after brain injury, and neurobehavioral abnormalities might be recovered if the behavioral tests are not performed shortly after brain injury^61^ and anxiety or cognitive neurobehavioral tests should be planned earlier in the model. Second, cognitive tests for the evaluation of learning and memory function in animal models are underdeveloped in terms of complexity, and most of the commonly used tests rely on stimuli and procedures, and this uncritical use of behavioral paradigms may account for the low predictability of rodent GMH models.^67^

This study has some limitations. First, this neonatal rat collagenase-induced GMH model only partially reproduces human preterm GMH and neurological sequelae. Therefore, the results obtained in this study should be extrapolated to humans with caution. Second, immune cells and related genes after hemorrhage were not fully classified and explored in the current study because of a lack of commercially available anti-rat antibodies for flow cytometry.

In conclusion, this study confirmed the early immune cell infiltration and neuroinflammation response in the immature brain after GMH. The animal model used here at least partially reproduces the GMH-induced brain injury, mild motor dysfunction, and locomotor hyperactivity seen in humans and therefore can be used as a valid tool in experimental studies of GMH-induced brain injury and in the development of effective therapeutic strategies. In addition, MRI is shown to be a valid tool for monitoring GMH-induced hematoma and secondary brain injury, therefore suggesting that MRI might be useful in evaluating preclinical intervention strategies for GMH-induced brain injury in the immature brain. Further study is needed to investigate the role of CD45^high^/CD11b^+^ cells in GMH-induced secondary brain injury and hematoma removal as well as the potential relationship between *Spp1* and *Lgals3* gene expression with CD45^high^/CD11b^+^ cell infiltration.
